# Avoiding initial hypothermia does not improve liver graft quality in a porcine donation after circulatory death (DCD) model of normothermic perfusion

**DOI:** 10.1371/journal.pone.0220786

**Published:** 2019-08-06

**Authors:** Jordan J. Nostedt, Tom Churchill, Sunita Ghosh, Aducio Thiesen, Jessica Hopkins, Mackenzie C. Lees, Benjamin Adam, Darren H. Freed, A. M. James Shapiro, David L. Bigam

**Affiliations:** 1 Department of Surgery, Division of General Surgery, University of Alberta, Edmonton AB, Canada; 2 Department of Surgery, Division of Surgical Research, University of Alberta, Edmonton AB, Canada; 3 Department of Mathematics and Statistical Sciences, University of Alberta, Edmonton AB, Canada; 4 Department of Laboratory Medicine and Pathology, University of Alberta, Edmonton AB, Canada; 5 Department of Physiology, University of Alberta, Edmonton AB, Canada; 6 Department of Biomedical Engineering, University of Alberta, Edmonton AB, Canada; 7 Department of Surgery, Division of Cardiac Surgery, University of Alberta, Edmonton AB, Canada; University of Colorado, Anschutz Medical Campus, UNITED STATES

## Abstract

**Background:**

Normothermic machine perfusion (NMP) of liver grafts donated after circulatory death (DCD) has shown promise in large animal and clinical trials. Following procurement, initial flush with a cold preservation solution is the standard of care. There is concern that initial cooling followed by warming may exacerbate liver injury, and the optimal initial flush temperature has yet to be identified. We hypothesize that avoidance of the initial cold flush will yield better quality liver grafts.

**Methods:**

Twenty-four anaesthetized pigs were withdrawn from mechanical ventilation and allowed to arrest. After 60-minutes of warm ischemia to simulate a DCD procurement, livers were flushed with histidine-tryptophan-ketoglutarate (HTK) at 4°C, 25°C or 35°C (n = 4 per group). For comparison, an adenosine-lidocaine crystalloid solution (AD), shown to have benefit at warm temperatures in heart perfusions, was also used (n = 4 per group). During 12-hours of NMP, adenosine triphosphate (ATP), lactate, transaminase levels, and histological injury were determined. Bile production and hemodynamics were monitored continuously.

**Results:**

ATP levels recovered substantially following 1-hour of NMP reaching pre-ischemic levels by the end of NMP with no difference between groups. There was no difference in peak aspartate aminotransferase (AST) or in lactate dehydrogenase (LDH). Portal vein resistance was lowest in the 4°C group reaching significance after 2 hours (0.13 CI -0.01,0.277, p = 0.025). Lactate levels recovered promptly with no difference between groups. Comparison to AD groups showed no statistical difference in the abovementioned parameters. On electron microscopy the HTK4°C group had the least edema with mean cell thickness of 2.92μm (p = 0.41) while also having the least sinusoidal dilatation with a mean diameter of 5.36μm (p = 0.04). For AD, the 25°C group had the lowest mean cell thickness at 3.14μm (p = 0.09).

**Conclusions:**

Avoidance of the initial cold flush failed to demonstrate added benefit over standard 4°C HTK in this DCD model of liver perfusion.

## Introduction

Liver transplant remains the only definitive therapy for end stage liver disease. However growing waitlist mortality has necessitated increased use of extended criteria [1] and donation after circulatory death (DCD) donors to address the shortage of transplantable livers.[2] Higher complication rates have been associated with use of DCD livers, most notably ischemic cholangiopathy.[3] The poor tolerance of prolonged static cold storage (SCS) for DCD liver grafts has led to recent growing interest in normothermic machine perfusion (NMP) as an alternative preservation strategy. Relative to SCS, NMP has shown improved preservation of DCD liver grafts in animal models[4–8] and promising results more recently in a large UK-European adequately powered randomized clinical trial.[9] As a result, this technology is progressing rapidly into the clinical realm. However the optimal perfusion strategy still remains a key focus for ongoing research. The initial liver flush before establishment of NMP represents an opportunity to further potentially optimize recovery and preservation of DCD liver grafts through controlled initial reperfusion. This is the first opportunity to intervene on the ischemic liver to affect optimal protection and recovery of function, and therefore warrants closer examination. The current clinical standard is to flush livers with a cold (4°C) preservation solution at the time of procurement and then store in cold preservation solution on ice until implantation. This practice of cold initial flush has continued in clinical trials where grafts are being preserved with NMP. Rapid cooling of organs before rewarming during NMP preservation has recently been found to be detrimental in experimental cardiac preservation. [10] DCD hearts that underwent initial flush with warm cardioplegic solution suffered less ischemia reperfusion injury and demonstrated improved functional recovery. These results were attributed to greater preservation of endothelial cell integrity at warmer flush temperatures.[10] This has not been explored previously to our knowledge in DCD porcine liver grafts.

The aim of this study is to determine if avoidance of hypothermia at the time of initial organ flush could improve liver graft quality for DCD liver grafts preserved using NMP. We hypothesize that avoiding hypothermia during initial organ flush for DCD liver grafts with prolonged warm ischemic time (WIT) will lead to overall improved graft preservation and function through better preservation of endothelial cell integrity as seen in DCD heart perfusions, and improved recovery of cellular ATP levels. Changing the temperature of the initial flush failed to demonstrate significantly improved liver graft quality overall, however when the temperature and solution composition were changed, different patterns of liver injury and hemodynamic parameters were observed which may warrant further investigation.

## Methods

Twelve DCD pigs were block randomized based on initial flush protocol. Histidine-tryptophan-ketoglutarate (HTK) solution (Servator H, Global Transplant Solutions, Ontario Canada), the standard clinical preservation solution, was used at 4°C (control), 25°C or 35°C (n = 4 per group). For comparison, using an additional 12 pigs, an adenosine-lidocaine crystalloid solution (AD) shown to have benefit at warmer temperatures in heart perfusions[10] was used at similar temperatures (n = 4 per group). Solution compositions are shown in **Table 1**. All animals received humane care in compliance with the National Institute of Health’s Guide for the Care of Laboratory Animals. The University of Alberta Animal Care and Use Committee approved the experimental protocol (AUP00001036).

**Table 1:** Composition of initial flush solutions.

	HTK (mmol/L)	AD (mmol/L)
Sodium chloride	15	123
Potassium chloride	9	5.9
Magnesium chloride	4	13
Histidine hydrochloride	18	
Histidine	180	
Tryptophan	2	
Mannitol	30	120
Calcium chloride	0.015	0.22
Potassium hydrogen 2-oxopentadioate	1	
Sodium bicarbonate		20
Sodium phosphate		1.2
Insulin		10 unit/L
Pyruvate		1
Reduced glutathione		3
Adenosine		0.4
Lidocaine		0.05
Glucose		10

### Liver procurement

Pigs were sedated with intramuscular injection of ketamine (20mg/kg) (Bimeda, Ontario, Canada) and anesthesia induced with inhaled isoflurane (Fresenius Kabi Canada Ltd, Ontario, Canada) with subsequent orotracheal intubation performed. General anesthesia was maintained with 2–3% isoflurane. A neck cut-down was performed for placement of an arterial pressure line in the left carotid artery for continuous pressure monitoring. A midline laparotomy was performed and a 10-gauge angiocath placed in the infra-renal vena cava for venous access. Hemodilution using 1 liter of Ringer’s Lactate was performed to allow for later extraction of blood to prime the NMP circuit. The porta hepatis was dissected to isolate the common bile duct, portal vein and common hepatic artery. A baseline liver parenchyma biopsy was obtained and then 500 units/kg of sodium heparin (Fresenius Kabi, Ontario, Canada) was administered. Approximately 800 ml of whole blood was extracted via the vena cava line and processed with a cell saver (Sorin Xtra, Germany). The washed red blood cells were used to prime the NMP circuit. Inspired oxygen was decreased to room air and the isoflurane increased to 5% to deepen anesthesia and then mechanical ventilation discontinued. Circulatory death was declared when a pulse on the arterial line waveform was no longer present and loss of cardiorespiratory activity confirmed with auscultation. The abdomen was closed using towel clips with intra-abdominal temperature measured throughout the warm ischemia period. A second liver biopsy was taken immediately following warm ischemia. The previously dissected vascular and biliary structures were divided and the liver taken to the back table for cannulation of the hepatic artery (RMI 8 French PED-A-008, Edwards Life Sciences, California, United States) and portal vein (Intersept Tubing Connector, Medtronic, Minnesota, United States).

**Fig 1:**
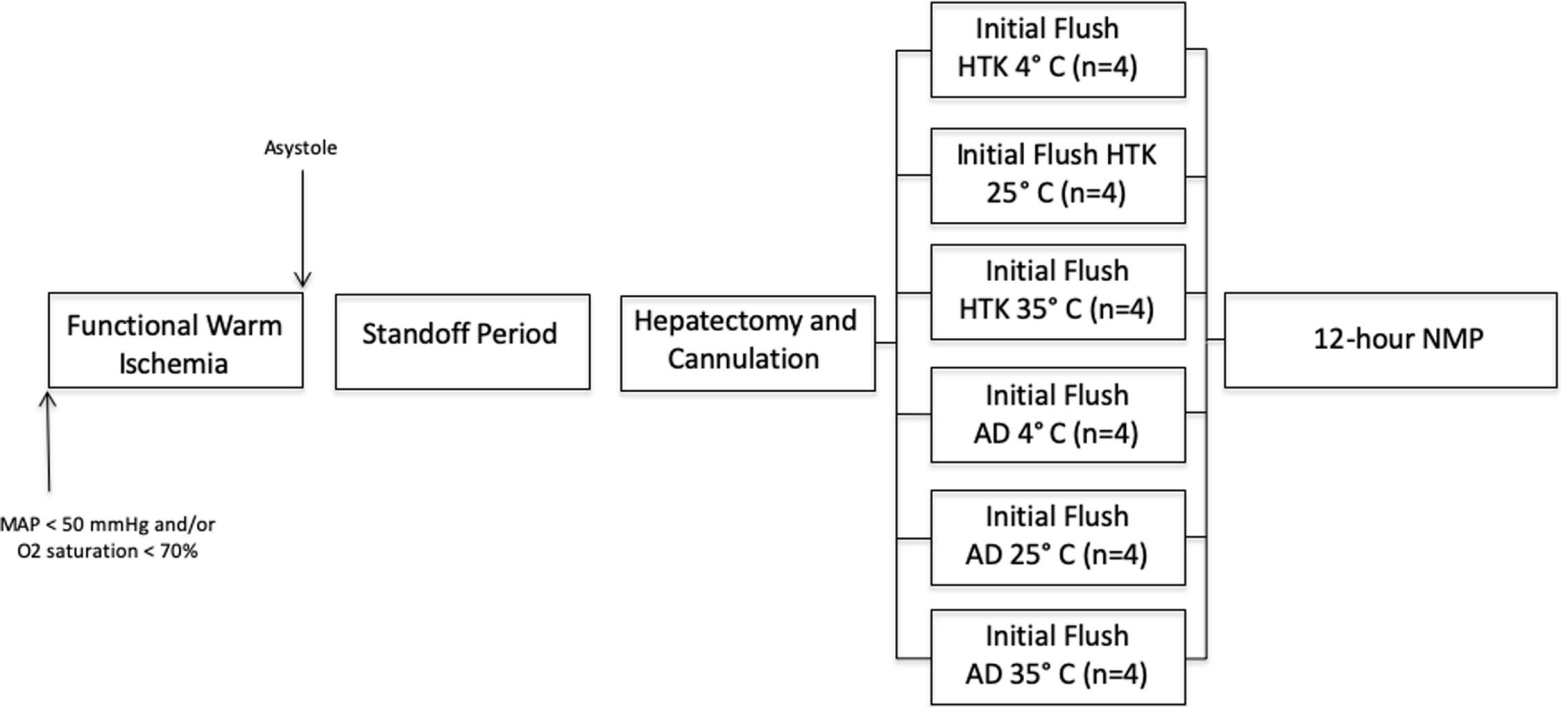
Experimental design. Total warm ischemic time was defined as the total time from when mean arterial pressure dropped below 50 mmHg or oxygen saturations were less than 70% to the initiation of the initial organ flush. Functional warm ischemia time was defined as the time from these criteria being reached to asystole. All experiments included a fixed 30-minute stand off period.

The WIT was approximately 60 minutes starting from the point where mean arterial pressure dropped below 50mmHg or oxygen saturation below 70% and extended to the onset of initial organ flush. The experimental design and warm ischemia timeline are summarized in **Fig 1.**

### Initial organ flush

The liver was flushed on the back table with dual perfusion through the hepatic artery and portal vein for 5 minutes. Temperature and composition of the flush solution was as per the groups described above. Temperature of the initial flush solution was controlled via a circuit that included a heat exchanger (Trillium Myotherm XP, Medtronic, Minnesota, United States), controlled water bath circulator (Poly Science PD07R-20-A11B, Illinois, United States), a centrifugal pump (BPX-80 Bi-Medicus, Medtronic, Minnesota, United States) and Affinity Fusion cardiotomy/venous reservoir (Medtronic, Minnesota, United States). During the 5-minute initial flush, temperature of the liver parenchyma was continually monitored. After the initial perfusion phase, livers were transferred to the primed NMP circuit and perfused continuously for 12 hours.

### Normothermic perfusion circuit

Perfusate consisted of Krebs-Henseleit based solution with albumin (glucose, sodium chloride, potassium chloride, calcium chloride, magnesium chloride, sodium bicarbonate, sodium phosphate and 8% bovine serum albumin) and autologous red blood cells in a 3 to 1 ratio. The circuit was primed with 3.375g Piperacillin-tazobactam (Sandoz, Quebec, Canada), 500mg methylprednisolone (Pfizer Canada, Quebec, Canada), and 10,000 units of sodium heparin (Fresenius Kabi, Ontario, Canada). Sodium bicarbonate 8.4% (Hospira, Quebec, Canada) was titrated to maintain pH of ≥7.30. Insulin was infused at 2 units/hour (NovoRapid, Novo Nordisk, Ontario, Canada). Glucose (Sigma Aldrich, Missouri, United States) was added as required based on hourly blood gas glucose readings titrating to 4-10mmol/L. Hepatic temperature was maintained at 37°C. Hepatic artery pressure was maintained at 60mmHg and the portal vein was perfused at a constant weight adjusted flow.

The experimental circuit was composed of a Dideco D764 cardiotomy reservoir (Dideco Inc. Italy) two BPX-80 Bi-Medicus centrifugal pumps (Medtronic, Minnesota, United States) for the hepatic artery and portal vein respectively, water bath circulator (Lab companion CW-05G, Jeio Tech, Korea) heat exchanger (Trillium Myotherm XP, Medtronic, Minnesota, United States) and Dideco D903 Avant Oxygenator (Dideco Inc. Italy). The vena cava flowed freely with venous outflow draining back to the reservoir. The bile duct was cannulated and bile production continuously monitored.

### Cellular energy levels

Biopsies were taken prior to the onset of WIT, following WIT, and at 1, 2, 4, 8 and 12 hours of NMP. Tissue samples were immediately frozen using Wollenberger clamps and liquid nitrogen then stored at -80°C. The frozen tissue was homogenized using 6% perchloric acid. Fifty microliters of homogenate was extracted and transferred to 0.15 M sodium hydroxide for measurement of protein content using the Lowry method as previously described.[11] The remaining homogenate supernatant was neutralized and ATP levels determined enzymatically.[12] Values were calculated in millimoles of ATP per gram of protein.

### Measurement of hepatocellular injury and inflammation

Alanine aminotransferase (ALT), aspartate aminotransferase (AST), and lactate dehydrogenase (LDH) were measured in the perfusate every 2 hours during NMP in our clinical laboratory using an enzymatic rate method on a Beckman Coulter DxC 800 analyzer (Beckman Coulter Canada, Ontario Canada). Inflammatory markers included tumor necrosis factor-α (TNF-α) and interleukin 6 (IL-6) levels in the perfusate, which were determined using porcine TNF-alpha and IL-6 ELISA kits (R&D Systems, Minnesota, United States). To further assess parenchymal injury a biopsy was taken at the end of dissection (pre-ischemia), and again at the end of 12 hours NMP. Samples were preserved in 10% formalin for hematoxylin and eosin (H&E) staining. H&E specimens were assessed by a blinded pathologist using light microscopy to assign injury score based on necrosis, hemorrhage, cholestasis and sinusoidal dilatation (**Table 2**).[13]

**Table 2:** Hepatocellular histologic injury scoring system[13].

Score	Hemorrhage	Necrosis	Cholestasis	Sinusoidal Dilatation
0	Absent	Absent	Absent	None
1	Focal	Peri-central	Present	Mild
2	Zonal	Zone 2+3	-	Moderate
3	Pan-lobular	Pan-lobular	-	Severe

### Liver function

Bile production was monitored continuously throughout perfusion. Tissue lactate was determined enzymatically.[12]

### Hemodynamics and endothelial injury

Pressure, flow and vascular resistance were continuously monitored for both the hepatic artery and portal vein.

Hyaluronic acid, a marker of sinusoidal endothelial cell injury[14], was measured using an ELISA kit (R&D Systems, Minnesota, United States). A random subset of biopsies taken after 12 hours of NMP (3 per group) was examined using electron microscopy for endothelial injury. Biopsies were fixed with a 2.5% glutaraldehyde/2% paraformaldehyde solution and then 1% osmium tetroxide. Samples were subsequently dehydrated and embedded in Spurr’s Resin. Two blocks from each sample (n = 6 per group) were sectioned and stained with uranyl acetate and lead citrate and photographed with a Philips Morgagni 268 transmission electron microscope (North American NanoPort, Hillsboro, Oregon, United States). A pathologist blinded to the experimental group assignment assessed maximum sinusoidal diameter, endothelial cell thickness at the nucleus and endothelial cell thickness at the thinnest point to determine endothelial cell edema. All measurements were taken perpendicular to the basement membrane.

### Statistical analysis

Graphs display data as mean values with standard error. ANOVA with Tukey Test for multiple comparisons was used to compare temperature groups for each solution. To compare all 6 groups, repeated measures ANOVA was used to include assessment of changes over time. Ordinal scale data was analyzed with the Kruskal-Wallis test. Statistical significance was defined as a p value of <0.05. Analysis was performed using SPSS (IBM Corp. Released 2013. IBM SPSS Statistics for Windows, Version 21.0. Armonk, NY: IBM Corp.).

## Results

### Warm ischemia time

The mean WIT was 62.4 minutes. Table 3 summarizes the experimental timeline for each experimental group. There was no difference in either total warm ischemic time (p = 0.75), or functional warm ischemic time (p = 95) between groups.

**Table 3:** Warm ischemic time.

Experimental Group	Mean Functional Warm Ischemic Time (SD)	Stand Off Period	Mean Hepatectomy Time (SD)	Mean Cannulation Time (SD)	Mean Total Warm Ischemic Time (SD)
HTK 4°C	13.3 (3.9)	30	9.3 (2.6)	7.3 (2.6)	66.7 (4.2)
HTK25°C	11.5 (9.3)	30	8.0 (1.4)	9.0 (2.9)	62.5 (9.8)
HTK35°C	11.0 (2.9)	30	7.8 (0.5)	7.3 (1.7)	58.3 (1.3)
AD4°C	15.5 (8.1)	30	6.8 (2.2)	7.3 (2.6)	62.0 (10.3)
AD25°C	13.3 (9.2)	30	6.8 (2.1)	9.3 (6.3)	61.3 (10.2)
AD35°C	13.3 (2.8)	30	8.3 (1.5)	8.0 (2.7)	64.3 (5.2)

All times are in minutes, SD: standard deviation.

### Cellular energy levels

**Fig 2:**
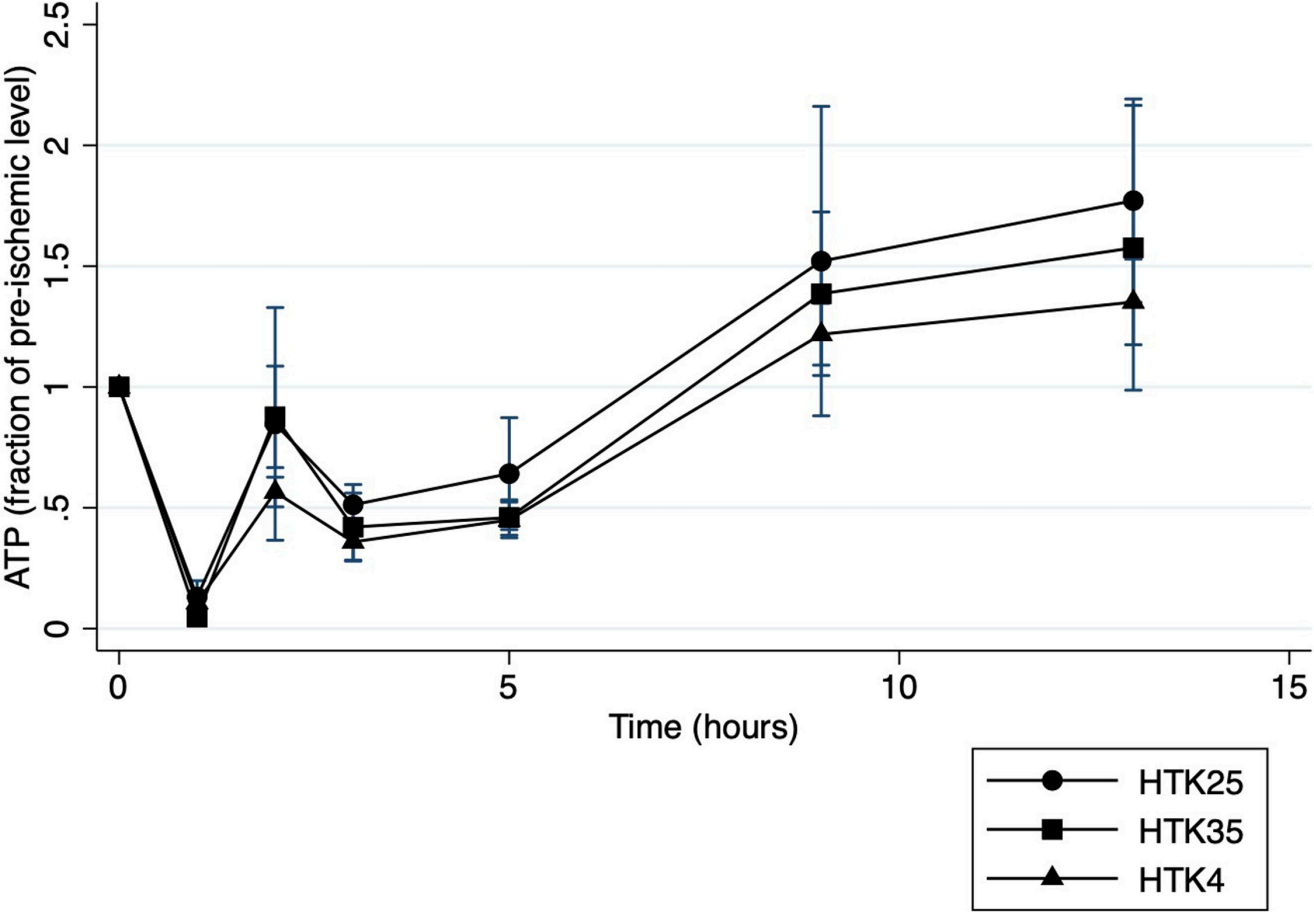
Tissue ATP during NMP. ATP levels during normothermic machine perfusion presented as a fraction of the pre-ischemia levels for histidine-tryptophan-ketoglutarate flushed livers. The first 2 time points represent pre-ischemia and post 60 minutes of warm ischemia respectively.

ATP levels as a fraction of pre-ischemia values are shown for the duration of the perfusion in **Fig 2**. Perturbations in ATP level were observed in all groups and found to be significant over time (P<0.001). There was a significant decrease following 60-minutes of WIT with partial recovery after 1 hour of perfusion followed by more gradual recovery to pre-ischemic levels, or higher by 8 hours of NMP. AD groups followed a similar pattern. There was no significant difference at any time point between temperature groups for any of the study solutions. When all groups were compared there was also no significant difference between groups (p = 0.88).

### Hepatocellular injury and inflammation

**Fig 3:**
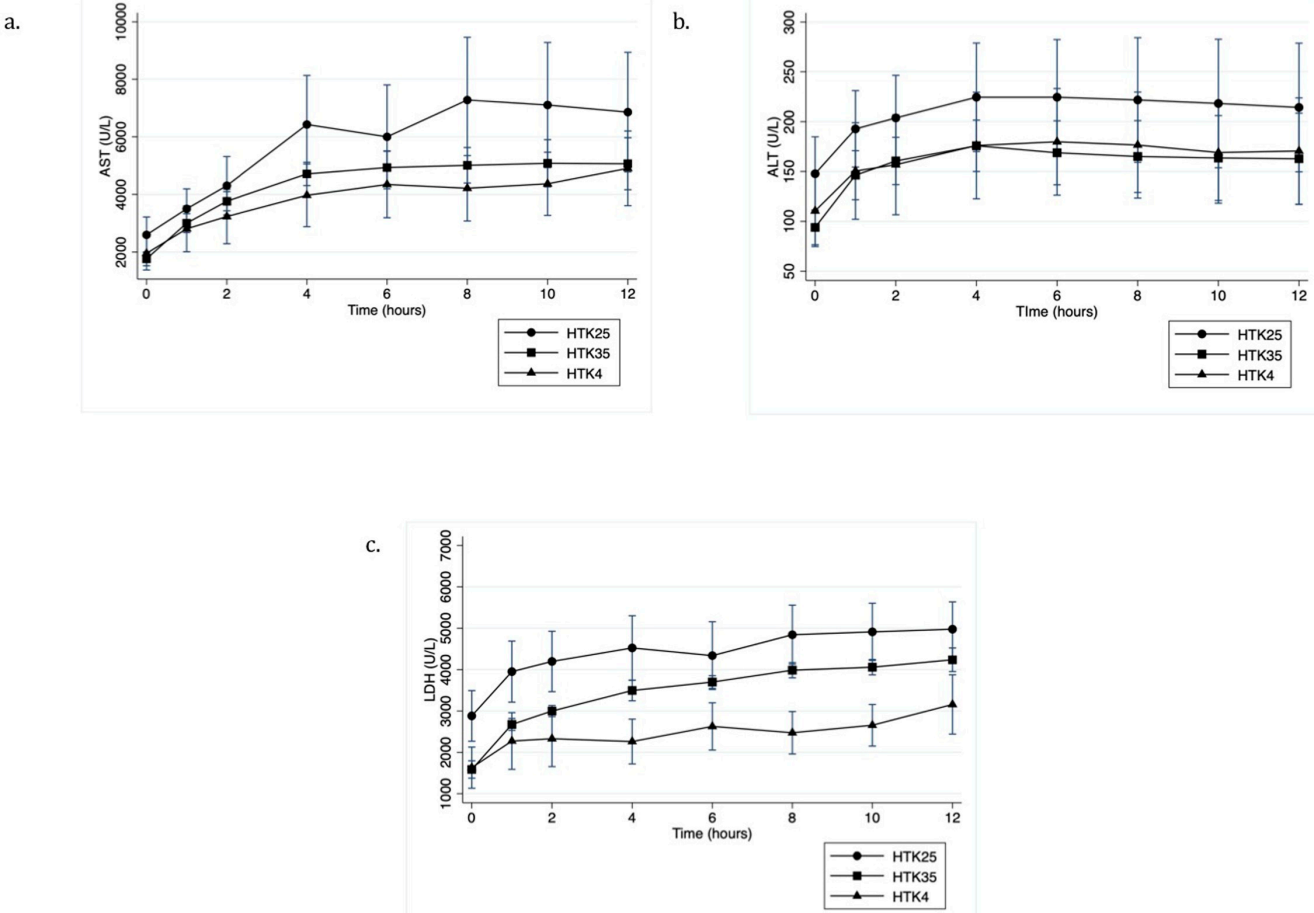
Hepatocellular injury markers during NMP. A) AST, B) ALT and C) LDH levels in the perfusate for histidine-tryptophan ketoglutarate flushed livers during NMP.

Hepatocellular enzyme levels in the perfusate are shown in **Fig 3**. In the HTK groups, the mean peak AST was lowest in the 4°C group although there was no statistically significant difference between groups (p = 0.51) (**Fig 3A)**. Peak ALT (**Fig 3B**) and LDH (**Fig 3C**) levels showed a similar pattern with no significant difference between groups for either peak ALT (p = 0.76) or peak LDH (p = 0.14). Though LDH was significantly lower at 4 (p = 0.04), 8 (p = 0.03) and 10 hours (p = 0.03) in the 4°C group relative to the 25°C, it did not reach significance relative to 35°C. For AD, the 4°C group showed the highest perfusate enzyme levels but no statistically significant difference between groups for peak AST (p = 0.53), ALT (p = 0.50) or LDH (p = 0.74). When all groups were compared there was no statistically significant difference in peak AST (p = 0.63), ALT (p = 0.80) or LDH (p = 0.31).

Histologic injury scores after 12 hours of NMP were lowest in the 4°C initial flush groups for both solutions. The HTK4°C and AD4°C groups scored 4.75 and 5 respectively on the 10 point injury scale (**Table 2**), where as the highest scores were seen in the HTK35°C and AD35°C groups at 6 and 6.25 respectively. This difference did not reach statistical significance (p = 0.78).

TNF-α levels were similar across temperatures in the HTK groups. For AD, the 4°C group had significantly lower levels of TNF-α in the first 2 hours of perfusion compared to both 25°C (p = 0.004) and 35°C (p = 0.002). When all groups were compared there was a significant difference between groups (p<0.001) with warm AD groups showing the highest levels, while IL-6 also showed elevated but non-significant levels in the warm AD groups (p = 0.08).

### Liver function

**Fig 4:**
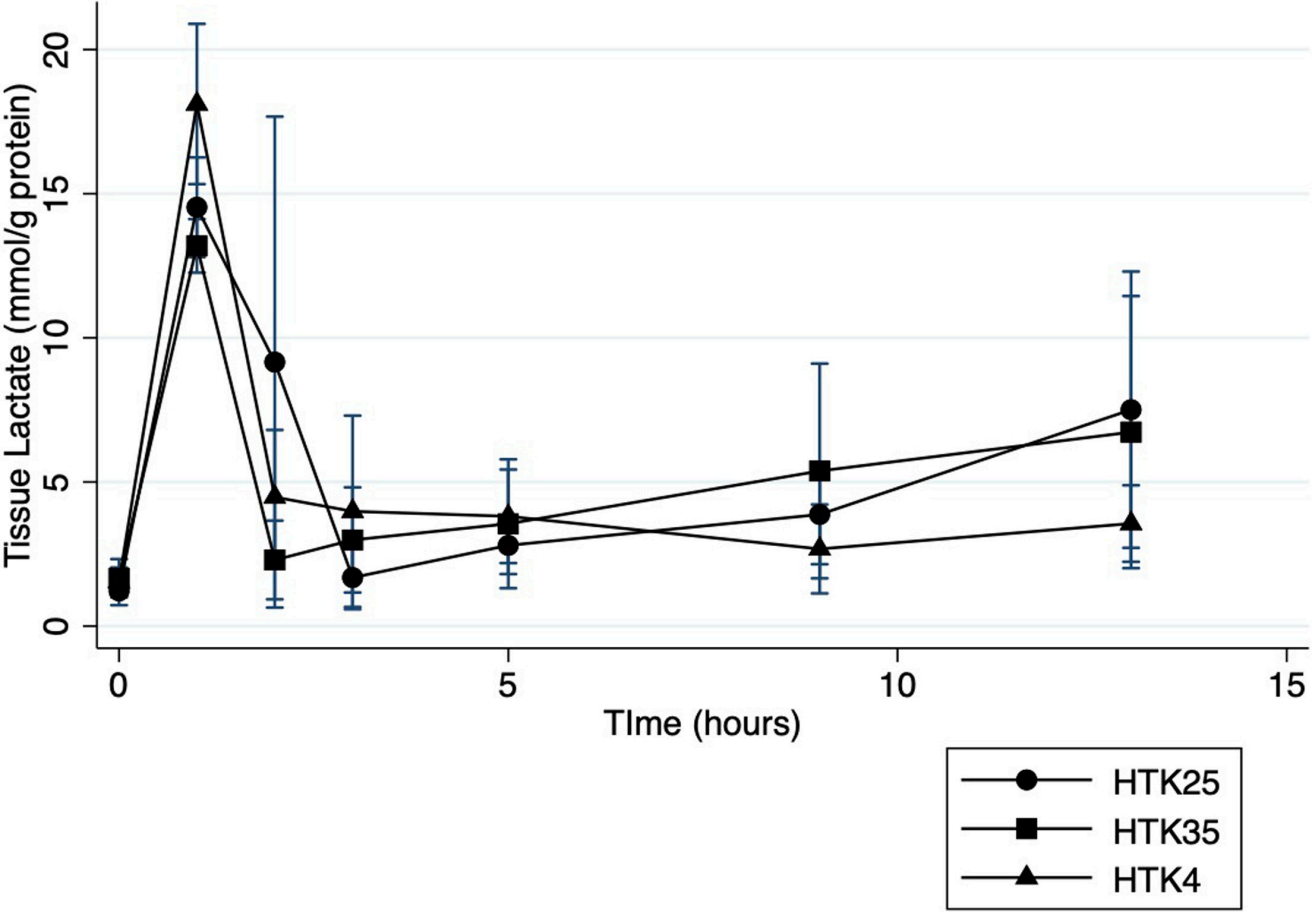
Tissue lactate during NMP. Lactate levels measured enzymatically from tissue biopsies during normothermic machine perfusion preservation of histidine-tryptophan-ketoglutarate flushed livers.

Tissue lactate levels changed significantly over time in all groups (p<0.001) with a significant rise following 60 minutes WIT (**Fig 4**). Lactate was cleared rapidly in the first 2 hours of perfusion for all groups and remained low for the duration of perfusion in all of the HTK and AD groups with no differences between temperatures for either solution. There was also no statistically significant difference when all groups were compared (p = 0.95).

The cold initial flush groups HTK4°C and AD4°C had the highest mean bile production of 117 grams (95% CI 0, 234) and 171 grams (95% CI 63, 279) over the 12 hours of NMP respectively, however there was no statistically significant difference between groups (p = 0.67).

### Hemodynamics and endothelial injury

**Fig 5:**
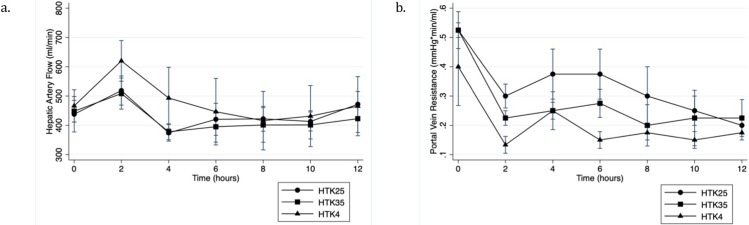
Hemodynamic parameters during NMP. A) Hepatic artery flow and B) Portal vein resistance during normothermic machine perfusion of histidine-tryptophan-ketoglutarate flushed livers.

Hepatic artery pressure was maintained at 60 mmHg and the arterial flow showed a similar pattern across all 6 groups with an initial increase over the first 2 hours of perfusion. Flows then decreased to reach steady flows by hour 6 for the remainder of the perfusion (**Fig 5A**). The 4°C group showed initially higher flows for HTK but similar steady state flows to the warm groups were reached by 6 hours. Statistically there was no significant difference noted between temperature groups at any time point. The AD4°C group had lower flows throughout the perfusion relative to the AD25°C and AD35°C groups though this difference did not reach statistical significance at any time point. When all 6 groups were compared there was no statistically significant difference between groups (P = 0.60).

Portal vein flow was kept constant. Portal vein resistance decreased significantly over the first 2 hours of perfusion (p<0.001) and remained stable for the remainder of the 12-hour perfusion (**Fig 5B**). For HTK, portal vein resistance was lowest in the 4°C group, reaching statistical significance at 2 hours of perfusion relative to 25°C (p = 0.03) but not statistically significant relative to 35°C (p = 0.21). For AD, the 25°C group had the lowest portal vein resistance throughout the perfusion though this did not reach statistical significance at any time point. When all groups were compared there was no statistical difference between groups (p = 0.26).

**Fig 6:**
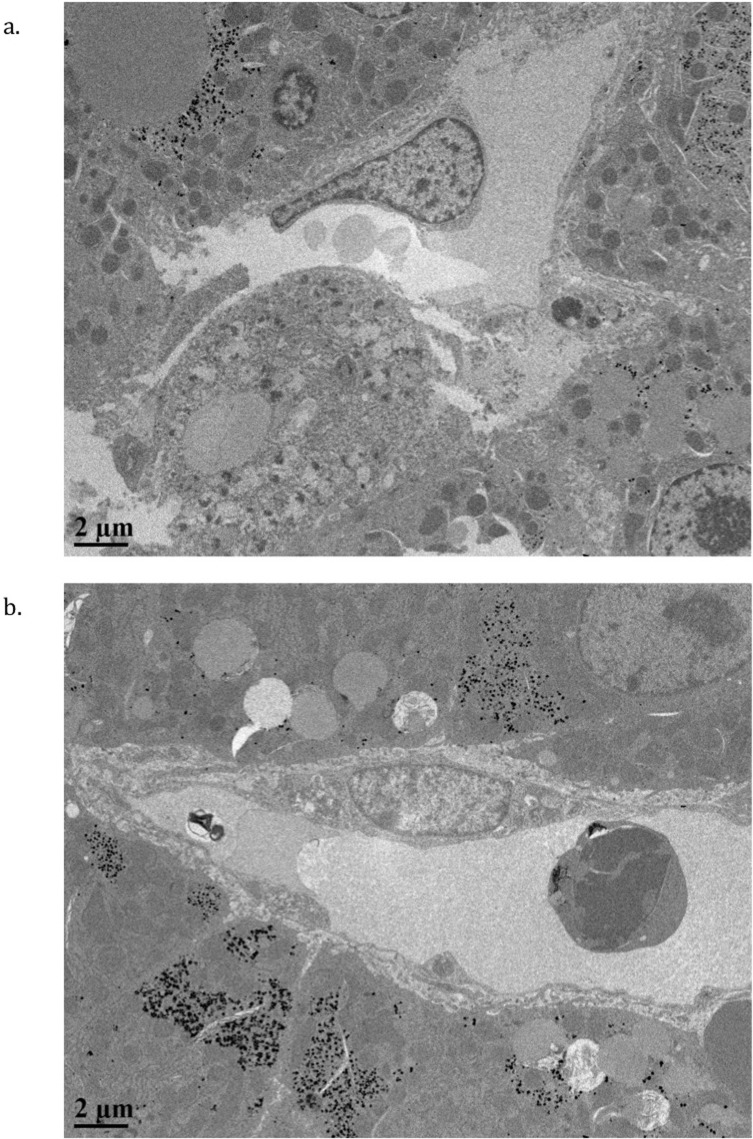
Electron micrographs of endothelial cells. Smaller sinusoidal luminal diameter was seen in HTK4°C groups (A), compared with HTK35°C groups (B).

Electron microscopy demonstrated a significant difference in sinusoidal dilatation between the HTK temperature groups (**Fig 6**) with the largest diameter seen in the 35°C group (7.60μm 95% CI 5.70, 9.5, p = 0.04). There was no statistically significant difference in endothelial cell thickness (p = 0.40) (**Table 4)**. In the AD groups there was no statistically significant difference in sinusoid diameter (p = 0.28) though endothelial cell thickness trended higher in the 4°C group (p = 0.09) (**Table 4)**.

**Table 4:** Electron microscopy assessment of sinusoidal endothelial cells. Maximum sinusoidal diameter, endothelial cell thickness at the nucleus and endothelial cell thickness at the thinnest point were measured to assess endothelial cell edema. All measurements were taken perpendicular to the basement membrane.

	Thickest/nucleus measurement (μm)	Thinnest point measurement (μm)	Sinusoidal max diameter (μm)
Histidine-tryptophan-ketoglutarate 4°C	2.92	0.14	5.36
Histidine-tryptophan-ketoglutarate 25°C	3.30	0.14	6.23
Histidine-tryptophan-ketoglutarate 35°C	3.22	0.15	7.60
p Value	0.41	0.90	0.04*
Adenosine-lidocaine solution 4°C	4.57	0.12	8.77
Adenosine-lidocaine solution 25°C	3.14	0.19	5.38
Adenosine-lidocaine solution 35°C	3.71	0.16	5.04
p Value	0.09	0.12	0.28

* Denotes statistical significance p<0.05

There was no significant difference in hyaluronic acid levels in the perfusate between groups at any time point.

## Discussion

DCD liver grafts represent a large potential resource for addressing the organ shortage currently facing liver transplantation. The results of recent clinical trials have expedited delivery of NMP technology in clinical liver transplantation. However optimal NMP protocols, especially for DCD grafts remain an ongoing target for research. Warm ischemia followed by immediate cooling endured by DCD grafts has been shown to be detrimental and leads to worse transplant outcomes.[15] Avoidance of initial cooling before establishment of NMP has not been investigated previously in DCD liver grafts to our knowledge, but has been shown to be beneficial in experimental cardiac preservation.[10] Our study in a large animal model suggests that altering the HTK flush temperature alone is insufficient to improve DCD liver graft quality. However warm initial flush with alternative solution compositions may indeed warrant further investigation.

After withdrawal of life support, DCD livers are exposed to a period of WIT where cells quickly shift to anaerobic metabolism. However at normothermic temperatures ATP consumption rapidly outpaces ATP production leading to depletion of cellular ATP stores.[16] This was confirmed in our DCD model with low ATP levels following 60 minutes of WIT, and is consistent with previous studies.[17] Maintenance of normal ion gradients across the cell membrane is vital for cellular function and is dependent upon the membrane sodium-potassium ATPase which is impaired by these low ATP levels.[18] Altered ionic flows ultimately lead to calcium influx, cellular edema and overall damage of cells priming them for further injury upon reperfusion.[18, 19] ATP levels in the peri-transplant period have been correlated with clinical transplant outcomes, with early allograft dysfunction.[20] Rapidly cooling the liver during initial flush reduces metabolic demands of the organ for subsequent SCS in accordance with the Q10 effect where each 10°C reduction in temperature is accompanied by a 1.5–2.5 fold drop in metabolic activity.[21] During SCS the reduced metabolic rate slows down ATP depletion but levels continue to decline during SCS resulting in severely depleted levels at the time of reperfusion.[22]

NMP has been shown in animal models to replenish cellular ATP levels in DCD liver grafts following WIT to levels nearly 80% of pre-ischemic values by 4 hours of perfusion.[17] Initial flush represents an important transition phase in the timeline of DCD liver grafts as they move from warm ischemia, to re-oxygenation on the NMP circuit. Reddy *et al*. found that even a brief one-hour period of SCS before NMP negated some of the positive effects of this modality.[23] SCS is eliminated in the setting where organs can be placed directly on the NMP circuit at the donor site as observed in clinical case reports. [9, 24–26] In this setting the period of initial flush is very short and the resulting negative effects of hypothermia may outweigh the benefit of a reduced metabolic rate for such a short period.[10] We hypothesized that rapid cooling during initial flush would impair the ability of the organ to recover cellular energy levels relative to liver grafts that were not exposed to hypothermic conditions. However, all of the groups in our study demonstrated significant recovery of ATP levels within the first hour of NMP, with ongoing recovery to pre-ischemic values by the end of the NMP period with no clear difference between temperatures for either HTK or AD. Additionally, comparing HTK and AD groups did not demonstrate a difference in ATP levels despite the presence of adenosine in AD, which has previously shown benefit for ATP levels in liver ischemia reperfusion injury.[27] The ATP results from this study confirm nearly total cellular ATP depletion following 60 minutes of WIT but also further validate previous studies [17] demonstrating NMP is capable of restoring ATP levels prior to transplantation. ATP levels were not significantly different between groups suggesting warm initial flush strategies with either HTK or AD provide no additional benefit over the current practice of cold HTK with respect to recovering cellular ATP levels for DCD grafts being preserved with NMP.

During WIT the depletion in ATP levels and the resultant disruption of membrane ionic gradients lead to hepatocyte damage that can be quantified by the measurement of transaminase release in the perfusate with transaminase levels during perfusion correlating with post transplant levels.[28] Kupffer cells in the liver clear transaminases from the circulation after they are released from hepatocytes.[29] A continuous rise in transaminase levels during NMP has been shown to predict poor graft viability.[30] Therefore the enzyme pattern during NMP gives an indication of the degree of hepatocellular damage, but also the recovery of the liver graft. Altering the initial flush strategy in our study did not significantly change the peak transaminase levels or the overall perfusate transaminase pattern over time with all groups demonstrating steady or declining transaminase levels by approximately 4 hours of perfusion. Enzyme levels in the HTK experiments appeared to be lower in the 4°C group, particularly LDH, while the 4°C initial flush seemed to have a pattern of higher enzyme levels in the AD groups. Reactive oxygen species (ROS) play a significant role in ischemic reperfusion injury. The AD solution contains reduced glutathione, absent from HTK; it plays a role in clearing ROS and has shown to be protective from ischemia reperfusion injury.[27] At cold temperatures during the transition to NMP where the tissue is first re-exposed to oxygen, glutathione peroxidase, which uses glutathione as a substrate to clear ROS, will function at a reduced capacity and thus, the benefit of this additive may be limited. That being said, we were underpowered to detect a significant differences when all groups were compared, and comparison of HTK4°C, AD25°C and AD35°C with larger numbers may be warranted to further assess the degree of hepatocellular injury with these novel strategies relative to the current clinical standard.

Of note, the warm AD groups did demonstrate higher TNF-α levels, which is suggestive of a higher degree of kupffer cell activation.[31] This would potentially place these grafts at risk for more significant ischemia reperfusion injury following reperfusion, however in the absence of a transplant phase this cannot be confirmed by this study.

Recovery of hepatocellular function was not different between the initial flush strategies tested. Lactate levels have consistently been used as a marker of liver function during *ex-situ* perfusion and have been included as a key component of proposed clinical viability criteria for liver grafts preserved with NMP.[32] During WIT reliance on anaerobic metabolism causes lactate levels to increase significantly as seen in our DCD model. Once NMP is initiated, rapid lactate clearance to within the normal range has been suggested to correlate with recovery of liver function and predict favorable transplant outcomes even in marginal grafts.[32] Our DCD model led to significant lactate accumulation following WIT followed by rapid clearance to low levels after only 1–2 hours of NMP. This pattern was consistently observed regardless of the initial flush strategy used, suggesting that altering the temperature of the brief initial flush does not impact recovery of hepatic function during NMP.

Bile production during NMP was initially thought to be an important predictor of graft viability however more recent data has emphasized the variability of *ex-situ* bile production.[30] Although still included in proposed viability criteria[32] low or absent bile production does not seem to reliably predict graft outcomes.[9, 32] The variability within groups in our study was significant making it difficult to draw any meaningful conclusions with respect to the effect of initial flush strategy on bile production.

Proposed graft viability criteria also include flows greater than 150 ml/min for the hepatic artery, and 500ml/min for the portal vein during NMP with these values correlating to improved graft outcomes.[32] Portal vein flow was controlled in our study and thus the portal vein resistance, also suggested to be an important marker of subsequent post transplant viability[13], was monitored. Portal vein resistance was lowest in the control group, suggesting no added benefit of warm flush on portal resistance. Regarding arterial flow, all groups maintained hepatic artery flows higher than the abovementioned threshold. When looking at the AD groups, although not statistically significant there was a pattern of higher flows in the warm AD groups where as the same could not be said for HTK. The highest flows were seen in the HTK 4°C and AD25°C groups which were also the groups demonstrating the lowest degree of sinusoidal endothelial cell edema and sinusoidal dilatation on electron microscopy. The importance of sinusoidal endothelial cell preservation in machine perfusion is becoming increasingly recognized.[33] Endothelial damage can lead to microcirculatory dysfunction upon reperfusion[34] which is hypothesized to play a role in ischemic cholangiopathy pathophysiology.[35] Ischemic cholangiopathy is a unique and troubling complication of DCD grafts. It often presents in a delayed fashion up to six months after transplant and thus assessing whether the abovementioned changes in flow and endothelial cell preservation could contribute to reducing ischemic cholangiopathy would require a more complex longitudinal transplant model with a more focused assessment of biliary injury.

Our study has several limitations. The primary operator in our experiments was not blinded to the conditions of each experiment due to logistical reasons regarding the set up of each experiment. Additionally, the conclusions drawn from this study are limited to the preservation phase, as we did not include a simulated transplant, or orthotopic transplantation phase. This limits our ability to determine whether or not altering the initial flush strategy would make a difference following transplantation. Furthermore, our experimental perfusion circuit was created in our lab as described above. This perfusion setup differs in some ways from other experimental circuits and clinical set ups and thus may further limit the generalizability of these results. Additionally, DCD liver transplantation has commonly been limited by ischemic biliary cholangiopathy. As mentioned above, this complication tends to occur months after transplantation and is thus difficult to study in a large animal model. In a recent study where immediate cooling following WIT in DCD liver grafts was avoided by the use of normothermic regional perfusion Watson *et al*. were able to show a significant reduction in ischemic cholangiopathy.[36] Thus the impact of avoiding immediate hypothermia during initial flush on the bile ducts of DCD liver grafts preserved with NMP may warrant further investigation. There were also some potential differences in hepatocellular injury markers and hemodynamic parameters that we were underpowered to show in our large animal model.

## Conclusion

This study suggests that initial flush at warmer temperatures provides no added benefit relative to the current standard of care of initial cold flush with HTK or alternate standard preservation solutions. Although temperature alone may not significantly change graft quality, solutions such as AD at warmer temperatures, may warrant subsequent investigation in larger powered models in efforts to further optimize NMP for DCD liver grafts.

## Supporting information

S1 Appendix Experimental data. (XLSX)
